# Supplementary material for: Olfactory specificity regulates lipid metabolism through neuroendocrine signaling in Caenorhabditis elegans
Source: Nat Commun. 2020 Mar 19;11:1450. doi: 10.1038/s41467-020-15296-8 (PMC7081233; doi:10.1038/s41467-020-15296-8)
Supplement: Supplementary file 1 — Supplementary Information [file 41467_2020_15296_MOESM1_ESM.pdf]

Supplementary Information

OLFACTORY SPECIFICITY REGULATES LIPID METABOLISM THROUGH NEUROENDOCRINE SIGNALING IN *CAENORHABDITIS ELEGANS*

Mutlu *et al.*

correspondence to: wmeng@bcm.edu, ozseker@bcm.edu

This PDF includes:

- Supplementary Figures 1-7
- Supplementary Table 1

Other Supplementary Materials for this manuscript includes:

- Supplementary Data 1. Number of worms used in the assays
- Supplementary Data 2. *C. elegans* strains used in this study
- Supplementary Data 3. List of primers used in this study
- Source Data File

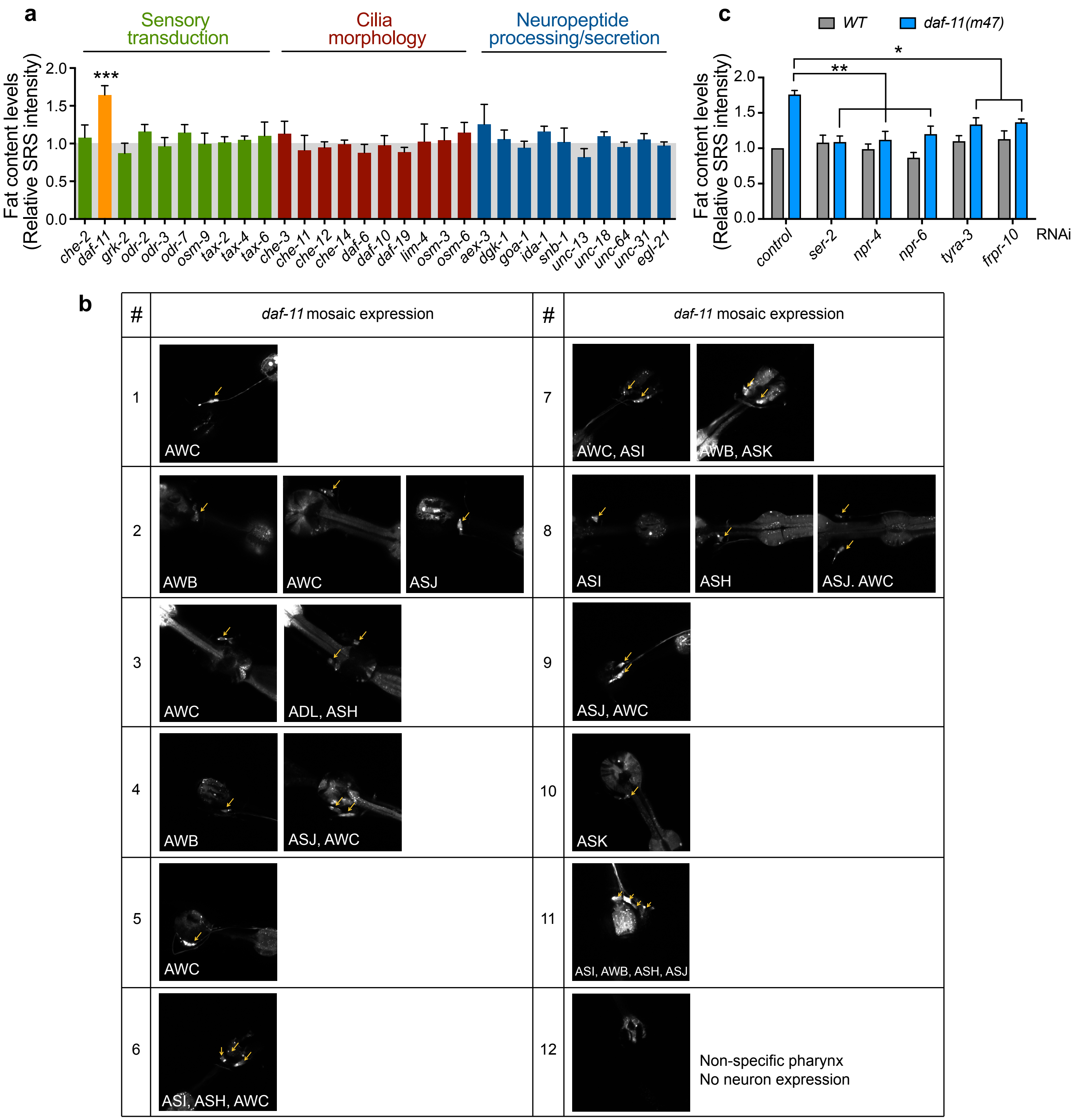

Supplementary Figure 1
Screens for chemosensory neurons and neuropeptide receptors that regulate peripheral fat storage.

a, Using SRS microscopy, fat content levels in the intestine, *C. elegans* fat storage tissue, were imaged and quantified in different neuronal chemosensory mutants at the L4 larval stage. Data are mean ± s.e.m., *** $P < 0.001$ by one-way ANOVA with Dunnett's multiple comparison test. **b**, Mosaic expression of *daf-11* in different chemosensory neurons in each individual worm is shown. Yellow arrows indicate the cell body of the neurons that *daf-11* is expressed in. **c**, Five neuropeptide GPCRs were discovered from RNAi screen, whose inactivation suppresses the increased fat storage level in the *daf-11* mutants. Data are mean ± s.e.m., * $P < 0.05$, ** $P < 0.01$ by two-way ANOVA with Sidak's multiple comparisons test. Numbers of animals used are listed in Supplementary Data 1. Source data for **a** and **c** are provided as a Source Data file.

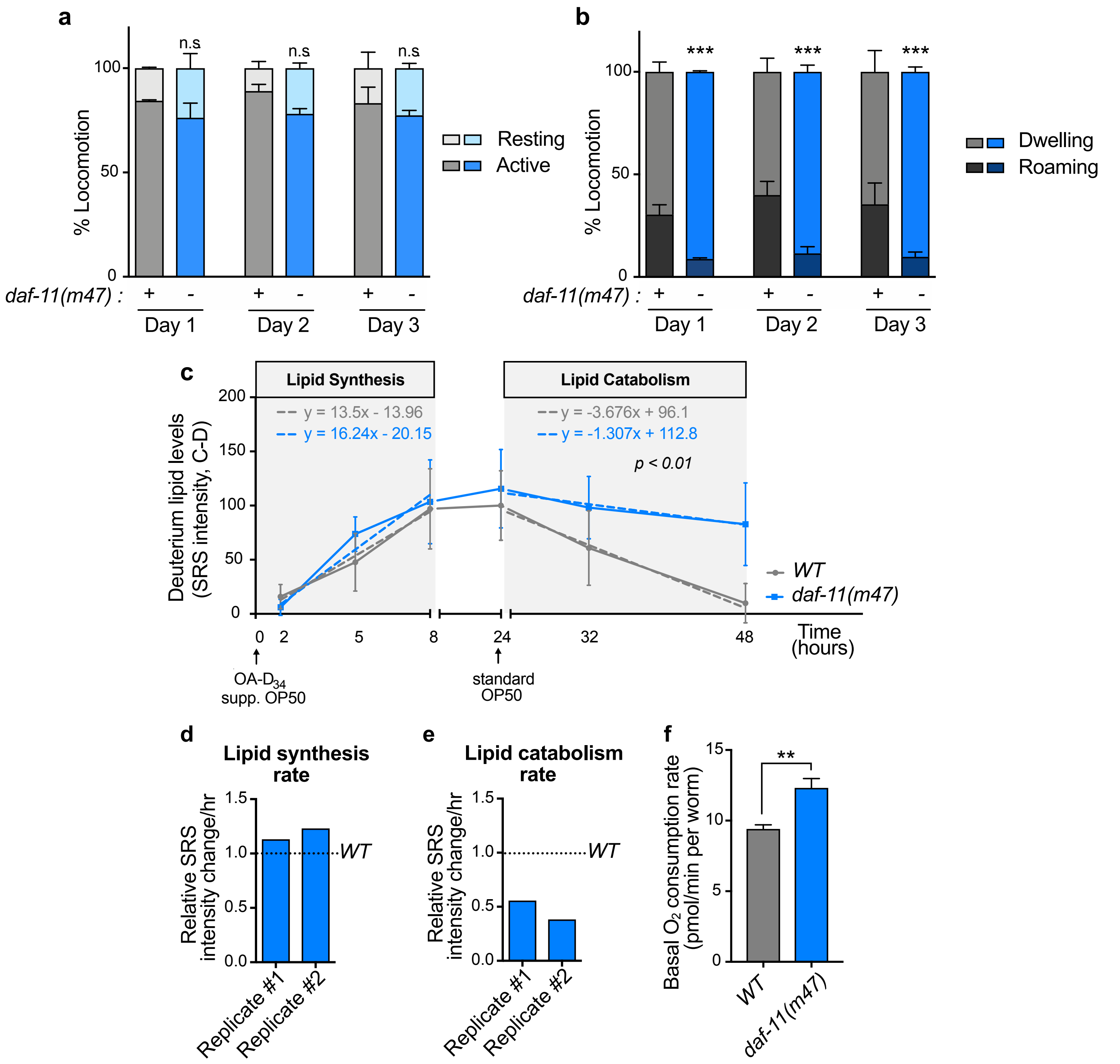

Supplementary Figure 2
Detailed analysis of energy utilization in *daf-11* mutants

a,b, Detailed analysis of locomotion indicates that in comparison to wild-type worms, *daf-11* mutants do not have significant changes in their active status. During their active time, *daf-11* mutants dwell more than they roam. From the locomotion trajectory analysis, “resting” refers to speed less than 5 μm/sec; “dwelling” represents speed higher than 5 μm/sec but less than 60 μm/sec and curvature more than 40° and “roaming” represents speed higher than 60 μm/sec and curvature less than 40°. Data are mean ± s.d., n=3 for each day and genotype, n.s., not significant, *** $P < 0.001$ one-way ANOVA with Tukey’s multiple comparison test. **c**, Signals derived from deuterium-labeled lipids were quantified at indicated time points using SRS microscopy, and compared between wild-type worms (WT) and the *daf-11* mutants. Dashed lines represent the trendlines and error bars represent s.d. For lipid catabolism $P < 0.01$ by linear regression analysis. **d,e**, Relative rates of lipid synthesis (**d**) and lipid catabolism (**e**) in the *daf-11* mutants in comparison to those in WT (shown in dashed lines) determined by linear regression analysis. **f**, Basal oxygen consumption rate (OCR) is measured using Seahorse extracellular flux analyzer. The *daf-11* mutants have an increased level of basal OCR compared to wild-type worms. Data are mean ± s.e.m. of three independent biological replicates, ** $P < 0.01$ by Student’s two-tailed t-test. Numbers of animals used are listed in Supplementary Data 1. Source data for **a-f** are provided as a Source Data file.

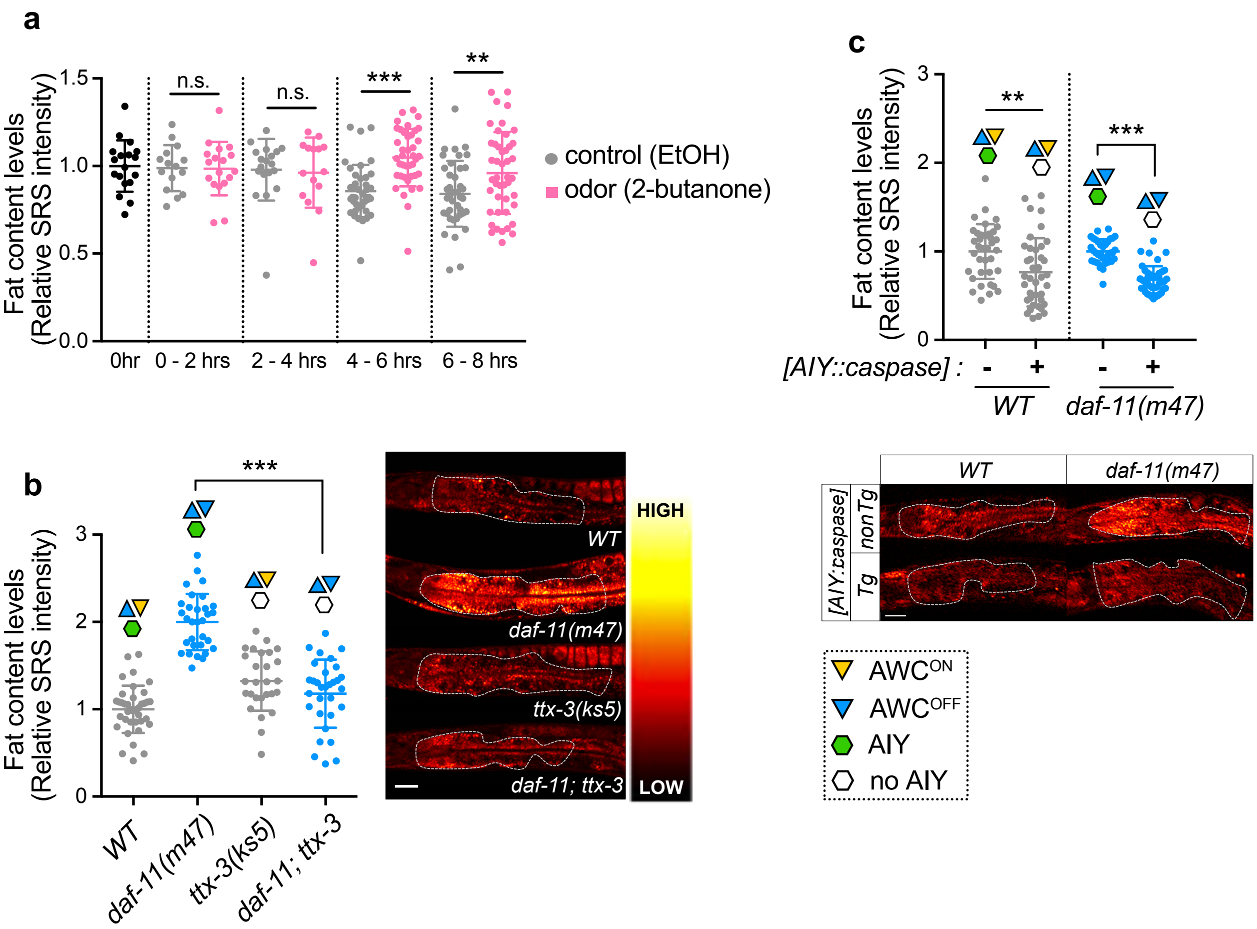

Supplementary Figure 3

2-butanone exposure reduces lipid catabolism, and AIY interneurons are required for the increased fat storage of the *daf-11* mutants.

a, 2-butanone exposure slows down lipid catabolism. Upon 4 hours of 2-butanone exposure, worms have significantly higher fat levels compared to worms that are exposed to the vehicle control, EtOH. **b**, Lack of AIY interneurons (by the ttx-3 mutation) suppresses the increased fat storage in the *daf-11* mutants. **c**, Genetic ablation of AIY interneurons (AIY::caspase) suppresses the increased fat storage in the *daf-11* mutants. Data are mean ± s.d., ** $P < 0.01$, *** $P < 0.001$, n.s. not significant by one-way ANOVA with Tukey's (**a,b**) and two-way ANOVA with Sidak's (**c**) multiple comparison test. Dashed lines indicate quantified intestine areas, yellow pixels indicate higher SRS signals. Numbers of animals used are listed in Supplementary Data 1. Source data **a-c** are provided as a Source Data file.

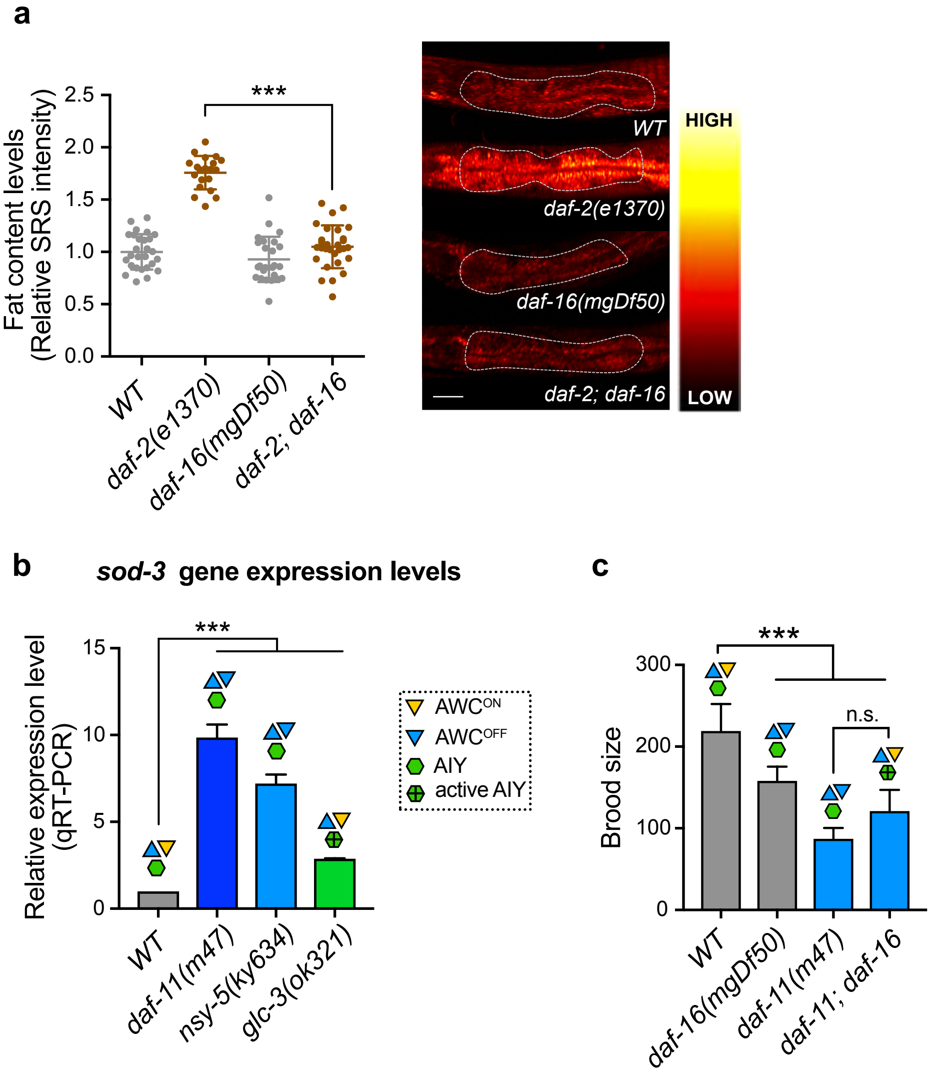

Supplementary Figure 4
DAF-16/FOXO regulates fat storage independently of its roles in dauer formation.

a, The *daf-16* deletion fully suppresses the increased fat storage in the *daf-2* mutants. Dashed lines indicate quantified intestine areas, yellow pixels indicate higher SRS signals. **b**, The expression of *sod-3*, the DAF-16/FOXO transcriptional target, is induced in the *daf-11* and *nsy-5* mutants with no AWC^{ON} neurons and in the *glc-3* mutants with activated AIY neurons. **c**, *daf-11* mutants have reduced brood size and *daf-16* inactivation does not suppress this decrease. For (**a**,**b** and **c**), data are mean ± s.d., *** $P < 0.001$, n.s. not significant by one-way ANOVA with Tukey's multiple comparison test. Numbers of animals used are listed in Supplementary Data 1. Source data for **a-c** are provided as a Source Data file.

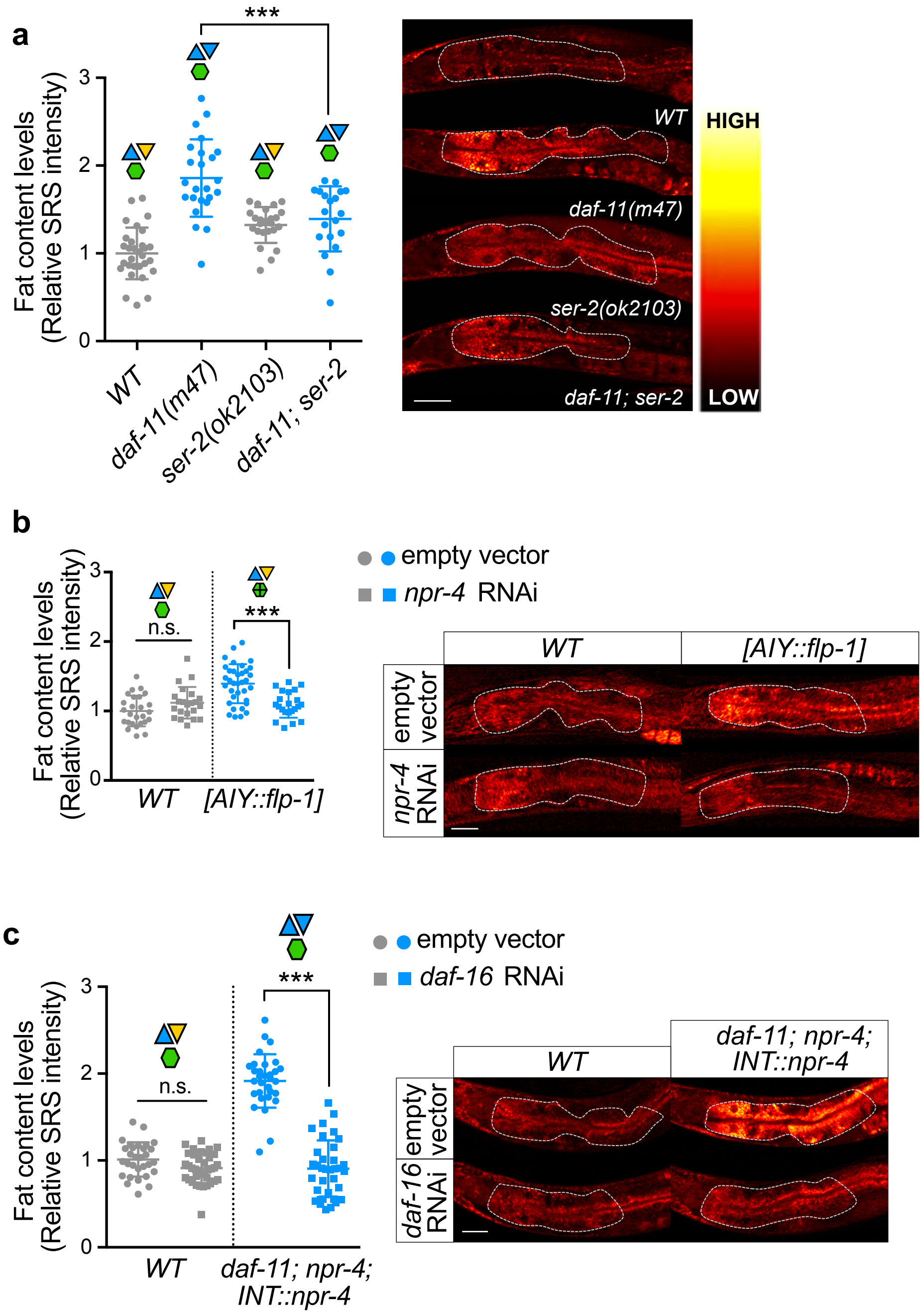

Supplementary Figure 5

Neuropeptide GPCR, *ser-2*, is required for the increased fat storage in the *daf-11* mutants, and *daf-16* acts downstream of *npr-4*.

a, Genetic deletion of *ser-2* suppresses the increased fat storage level in the *daf-11* mutants. **b**, *npr-4* knock-down by RNAi suppresses the fat storage increase in *flp-1* overexpressing transgenics. **c**, When its downstream transcription factor *daf-16* is knocked down by RNAi, NPR-4 restoration fails to rescue the fat storage increase in the *daf-11; npr-4* double mutants. (**a-c**) Data are mean ± s.d., *** $P < 0.01$, n.s. not significant by two-way ANOVA with Sidak's multiple comparisons test. Scale bar = 40μm. Dashed lines indicate quantified intestine areas, yellow pixels indicate higher SRS signals. Numbers of animals used are listed in Supplementary Data 1. Source data for **a-c** are provided as a Source Data file.

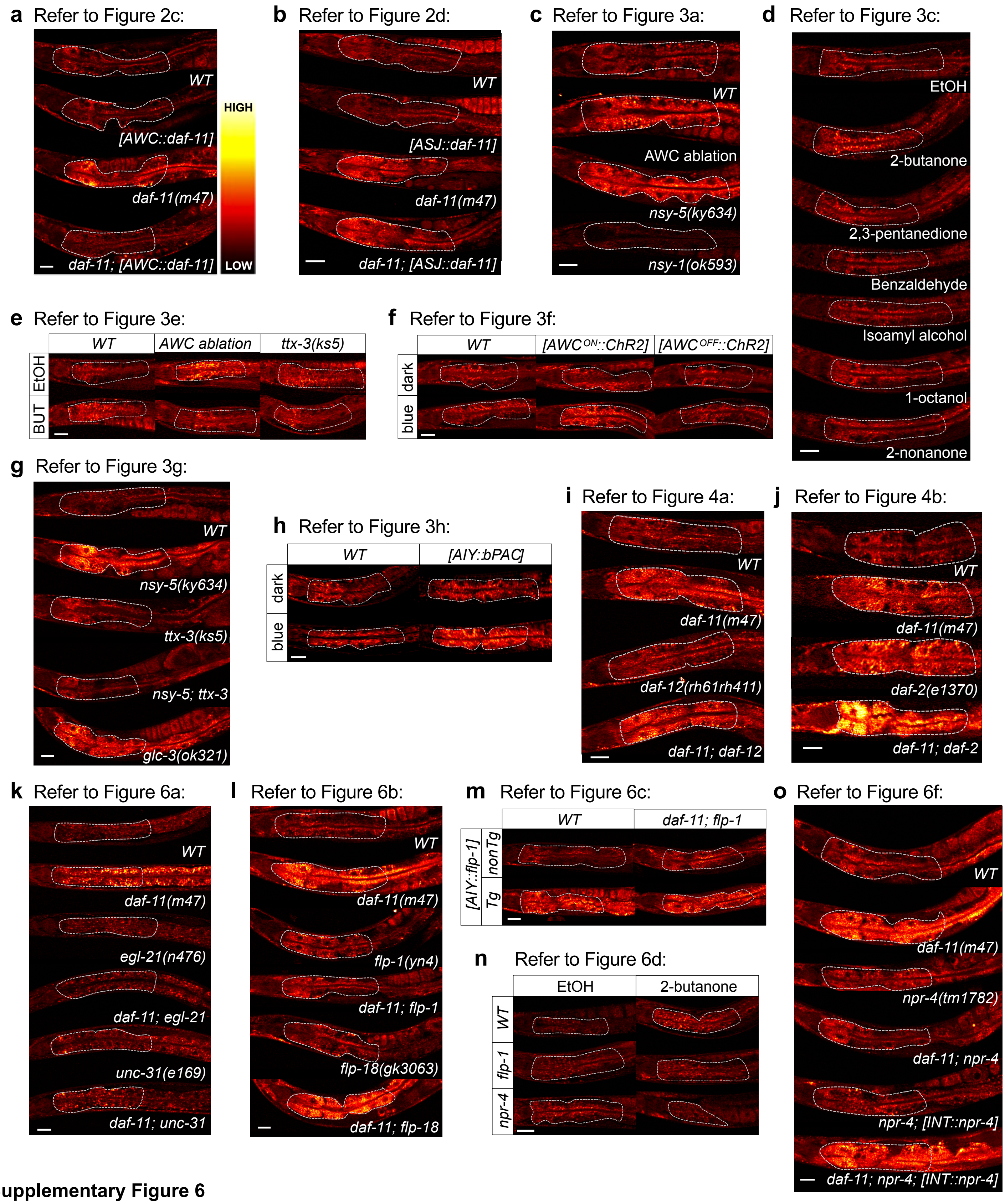

Supplementary Figure 6
Representative SRS microscopy images referring to the fat level quantification results in main figures.

a, Representative SRS images referring to Figure 2c. **b**, Representative SRS images referring to Figure 2d. **c**, Representative SRS images referring to Figure 3a. **d**, Representative SRS images referring to Figure 3c. **e**, Representative SRS images referring to Figure 3e. **f**, Representative SRS images referring to Figure 3f. **g**, Representative SRS images referring to Figure 3g. **h**, Representative SRS images referring to Figure 3h. **i**, SRS images referring to Figure 4a. **j**, Representative SRS images referring to Figure 4b. **k**, Representative SRS images referring to Figure 6a. **l**, Representative SRS images referring to Figure 6b. **m**, Representative SRS images referring to Figure 6c. **n**, Representative SRS images referring to Figure 6d. **o**, Representative SRS images referring to Figure 6f. **(a-o)** Yellow pixels indicate high SRS signals. Dashed lines indicate the quantified intestinal area. Scale bar = 40μm.

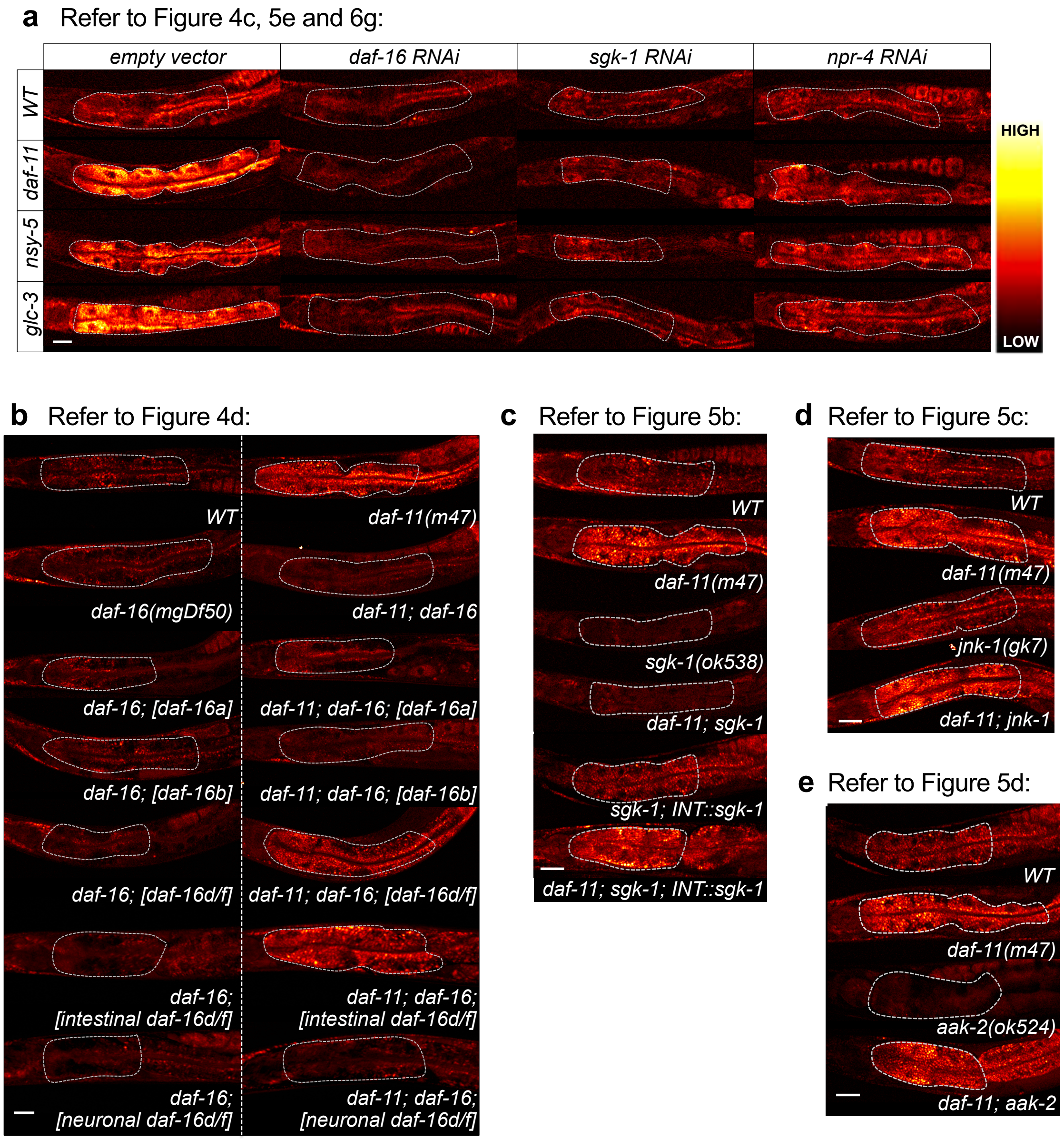

Supplementary Figure 7
Representative SRS microscopy images referring to the fat level quantification results in main figures (continued).

a, Representative SRS images referring to Figure 4c,5e and 6g. **b**, Representative SRS images referring to Figure 4d. **c**, Representative SRS images referring to Figure 5b. **d**, Representative SRS images referring to Figure 5c. **e**, Representative SRS images referring to Figure 5d. **(a-e)** Yellow pixels indicate high SRS signals. Dashed lines indicate the quantified intestinal area. Scale bar = 40µm.

RNAi	Relative fat content level	% change		RNAi	Relative fat content level	% change
control	1.833146864	-		F54D7.3	1.745025159	-4.81
AC7.1	2.754812882	+50.28		F56B6.5	1.754941974	-4.27
C02D4.2 (*ser-2*)	1.270420565	-30.70		F57A8.4	1.857532658	+1.33
C04E6.9	1.89757	+3.51		F57H12.4 (*frpr-10*)	1.260393101	-31.24
C09B7.1	2.047624677	+11.70		F59B2.13	1.684382975	-8.12
C10C6.2	1.745965196	-4.76		F59C12.2	1.819755187	-0.73
C15B12.5a	1.726873802	-5.80		F59D12.1	1.818832451	-0.78
C16D6.2 (*npr-4*)	1.42673145	-22.17		K07E8.5	1.548055233	-15.55
C26F1.6	1.855297017	+1.21		K09G1.4	1.865157612	+1.75
C30F12.6	1.764284495	-3.76		K10B4.4	1.909524839	+4.17
C38C10.1	1.732740517	-5.48		K10C8.2	1.871576268	+2.10
C39E6.6	1.773150811	-3.27		M03F4.3 (*tyra-3*)	1.368818902	-25.33
C43C3.2	1.74904122	-4.59		R106.2	1.738423757	-5.17
C44C3.5	1.7815588	-2.81		R12C12.3	1.941353249	+5.90
C48C5.1	1.678830433	-8.42		R13H7.2	1.573938216	-14.14
C49A9.7	1.850837911	+0.97		T02D1.4	1.602461194	-12.58
C50F7.1	1.916569926	+4.55		T02D1.6	1.647314769	-10.14
C52B11.3	1.805614632	-1.50		T02E9.1	2.03719025	+11.13
C53C7.1	1.662183711	-9.33		T05A1.1	1.687344875	-7.95
C54A12.2	1.701140584	-7.20		T07D10.2	1.624155088	-11.40
C56A3.3	1.986535125	+8.37		T07D4.1	1.613711224	-11.97
C56G3.1	1.771276258	-3.38		T11F9.1	1.688582138	-7.89
E04D5.2	1.823862797	-0.51		T14E8.3	1.876199676	+2.35
F01E11.5	1.702957078	-7.10		T19F4.1a	1.872622324	+2.15
F02E8.2	1.986610726	+8.37		T22D1.12	1.86836796	+1.92
F14D12.6	1.933176123	+5.46		T23B3.4	1.836676775	+0.19
F14F4.1	1.70850118	-6.80		T27D1.3	1.645294902	-10.25
F15A8.5	1.67106683	-8.84		W05B5.2	1.913083137	+4.36
F16C3.1	1.619120085	-11.68		Y116A8B.5	1.716910634	-6.34
F16D3.7	1.824578842	-0.47		Y39A3B.5	1.885891281	+2.88
F31B9.1	1.772600528	-3.30		Y40H4A.1	1.837757581	+0.25
F35G8.1	1.783551666	-2.71		Y41D4A.8	2.213711485	+20.76
F41E7.3 (*npr-6*)	1.401109445	-23.57		Y54E2A.1	1.962458486	-7.05
F42C5.2	1.863227075	+1.64		Y62E10A.4	1.914473507	-4.44
F47D12.1	1.682065774	-8.24		ZC412.1	1.792018339	-2.24
F53A9.5	1.780031431	-2.90		ZK455.3	1.63155899	-11.00

Supplementary Table 1
Neuropeptide GPCR RNAi screen in the *daf-11* mutant.

The “relative fat content level” in the *daf-11* mutants compared to WT for each individual GPCR knock-down is listed. Percent change in the fat content level (% change) is calculated by normalizing RNAi inactivation to the control with only empty vectors. Five GPCRs with more than 20% suppression were chosen as candidates highlighted in yellow.
