# Supplementary material for: Olfactory specificity regulates lipid metabolism through neuroendocrine signaling in Caenorhabditis elegans
Source: Nat Commun. 2020 Mar 19;11:1450. doi: 10.1038/s41467-020-15296-8 (PMC7081233; doi:10.1038/s41467-020-15296-8)
Supplement: Supplementary file 3 — Description of Additional Supplementary Files [file 41467_2020_15296_MOESM3_ESM.pdf]

Description of Additional Supplementary Files

File Name: Supplementary Data 1
Description: Supplementary Data 1 contains the numbers of worms for each genotype used in the assays and shown in the figure panels.

File Name: Supplementary Data 2
Description: Supplementary Data 2 contains the list of *C. elegans* mutants and transgenics used in this study including the ones obtained from Caenorabditis Genetics Center, National Bioresource Project and other labs, as well as others that were generated in our lab.

File Name: Supplementary Data 3
Description: Supplementary Data 3 contains the list of primers used in this study including genotyping primers used to backcross and to generate double mutants, as well as qRT-PCR primers.
